# A soy-based phosphatidylserine/ phosphatidic acid complex (PAS) normalizes the stress reactivity of hypothalamus-pituitary-adrenal-axis in chronically stressed male subjects: a randomized, placebo-controlled study

**DOI:** 10.1186/1476-511X-13-121

**Published:** 2014-07-31

**Authors:** Juliane Hellhammer, Dominic Vogt, Nadin Franz, Ulla Freitas, David Rutenberg

**Affiliations:** 1 Diagnostic Assessment and Clinical Research Organization (Daacro) GmbH & Co. KG, Science Park Trier, Max-Planck-Str. 22, 54296 Trier, Germany; 2 Lonza Ltd, Muenchensteinerstr. 38, 4002 Basel, Switzerland; 3 Lipogen Ltd, P.O.Box 7687, 31078 Haifa, Israel

**Keywords:** Phosphatidylserine, Phosphatidic acid, Memree, Stress, PAS, Cortisol, Acute stress, Chronic stress, Stress response, TSST

## Abstract

**Background:**

Supplementation with a phosphatidylserine and phosphatidylserine/ phosphatidic acid complex (PAS) has been observed to normalize stress induced dysregulations of the hypothalamus-pituitary-adrenal axis (HPAA). Prolonged stress first induces a hyper-activation of the HPAA, which then can be followed by a state of hypo-activation.

The aim of this study was to examine effects of an oral supplementation with 400 mg PS & 400 mg PA (PAS 400) per day on the endocrine stress response (ACTH, saliva and serum cortisol) to a psychosocial stressor. A special focus was to analyze subgroups of low versus high chronically stressed subjects as well as to test efficacy of 200 mg PS & 200 mg PA (PAS 200).

**Methods:**

75 healthy male volunteers were enrolled for this double-blind, placebo-controlled study, stratified by chronic stress level, and randomly allocated to one of three study arms (placebo, PAS 200 and PAS 400 per day, respectively). Study supplementation was administered for 42 days for each participant. Chronic stress was measured with the Trier Inventory for Chronic Stress (TICS), and subgroups of high and low chronic stress were differentiated by median values as provided by the TICS authors. A six week period of supplementation was followed by an acute stress test (Trier Social Stress Test - TSST).

**Results:**

Chronic stress levels and other baseline measures did not differ between treatment groups (all p > 0.05). Acute stress was successfully induced by the TSST and resulted in a hyper-responsivity of the HPAA in chronically stressed subjects. Compared to placebo, a supplementation with a daily dose of PAS 400 was effective in normalizing the ACTH (p = 0.010), salivary (p = 0.043) and serum cortisol responses (p = 0.035) to the TSST in chronically high but not in low stressed subjects (all p > 0.05). Compared to placebo, supplementation with PAS 200 did not result in any significant differences in these variables (all p > 0.05). There were no significant effects of supplementation with PAS on heart rate, pulse transit time, or psychological stress response (all p > 0.05).

**Conclusion:**

In chronically stressed subjects, a supplementation with PAS 400 (MemreePlus™) can normalize the hyper-responsivity of the HPAA to an acute stressor.

**Trial registration:**

Trial registration: DRKS-ID: DRKS00005125

## Background

Stress is an individual response to external and internal challenges ranging from behavioral to molecular adaptions, affecting the stress response network in the central nervous system and its crosstalk with peripheral organs via the endocrine and the autonomic nervous systems [1]. The hypothalamic-pituitary-adrenal axis (HPAA) plays a central role in such adaptation processes of the organism [2]. In response to acute stressors, the hypothalamus secretes corticotropin–releasing factor, which triggers the release of adrenocorticotropic hormone (ACTH) from the pituitary gland. ACTH then promotes the release of cortisol from the adrenal cortex. Cortisol affects numerous physiological functions, such as carbohydrate, lipid and protein metabolism, the immune system, and enhances the energy supply of the central nervous system. Chronic psychological stress has been shown to induce dysregulations of the HPAA, which are associated with impaired psychological and somatic well-being. Under such conditions, a biphasic response of the HPAA has been observed: prolonged psychological stress may first induce a hyper-activation of the HPAA, which may then be followed by a state of hypo-activation [3,4]. While a hyper-activation of the HPAA has been shown to be associated with stress related disorders such as melancholic depression, anxiety disorders, and metabolic syndrome, a hypo-activation is rather linked to fatigue, pain, irritability and associated disorders such as fibromyalgia, arthritis, and chronic fatigue syndrome [5-7]. Thus, a hyper-activation of the HPAA seems to be associated with a hyper-reactivity to acute stress, while a reduced synthesis of cortisol may mainly account for a hypoactive state [6,8-12].

Recent research showed that the intake of certain nutraceuticals reversed or normalized such dysregulations and improved stress symptomatology. For example, the intake of fertilized egg powder for the duration of four weeks normalized the psychological and physiological stress response in chronically stressed but otherwise healthy males [13]. Moreover, studies on different substances enriched with phospholipids have shown beneficial effects in chronically stressed subjects. It has been suggested that milk phospholipids may increase cortisol availability in subjects with high chronic stress [13-15].

Phosphatidic acid (PA) is one of the most important glycerophospholipids found in bio-membranes. PA has different roles in the cell: it is a precursor for other lipids such as phosphatidylserine (PS) or phosphatidylcholine via the conversion of PA to diacylglycerol [16]. Moreover, PA influences membrane curvature [17,18] and acts as signaling lipid [19,20]. Animal research suggests that PA may prevent or restore gastrointestinal disorders [21,22] and may increase skeletal muscle mass [23]. PS plays a key role in neuronal cell structure and functioning and may improve memory, learning, mood and stress management [24-29]. PS has been shown to decrease ACTH and cortisol responses to acute physical and mental stress [30-35]. Additionally, the intake of PS has been associated with an improvement of psychiatric disorders, such as bipolar and major depressive disorders reviewed in [26,36], as well as with the prevention of inflammatory neurodegenerative events [37].

Moreover, in combination with PS, PA has been shown to reduce cortisol levels and enhance well-being under acute social stress. In a previous study, we could show that the administration of PAS 400 per day over a period of 21 days resulted in both a significant decrease of the ACTH- and cortisol-response, and, in addition, a reduction of anxiety to the TSST [27]. The exact mechanisms on how PA and PS contribute to an adaptation of the stress response network are still unknown. Several stress-related disorders such as depression and mood disorders have been associated with fatty acid deficiencies [36,38-41]. The intake of dietary phospholipids such as PA and PS may cause alterations and optimize the composition of cell membranes which in turn may modulate cellular function and activity of membrane [42].

The aim of the current study was to confirm the HPAA normalizing effects of a dose of PAS 400 found in a previous study. Here, we were especially interested whether there was a difference in effects between chronically stressed or non-stressed people. Chronic stress was assessed by using the screening scale for chronic stress of the Trier Inventory for Chronic Stress (TICS) [43]. Cut-off for low versus high chronic stress was defined as provided by a norm population of the TICS authors. In addition, we wanted to investigate whether these effects can also be observed with a lower dosage of PAS 200. In order to investigate PAS effects in a homogeneous study population, we included only male subjects and enlarged group size. We investigated the effects of two different doses of PAS (200 mg PA & 200 mg PS and 400 mg PA & 400 mg PS per day) over 42 days compared to placebo on psychological and physiological stress reactivity in subjects stratified by low and high chronic stress levels.

## Results

### Baseline measures

Groups did not differ with respect to age, height, body mass, BMI (body mass index), WHR (waist to hip ratio), systolic blood pressure, diastolic blood pressure and heart rate (Table 1). Furthermore, no baseline differences were observed for measures of chronic stress (Trier Inventory of Chronic Stress; TICS; all p > 0.05; data not shown) between all three treatment groups.

**Table 1:** Baseline measures

	**Age [years]**	**Height [m]**	**Body mass [kg]**	**BMI [kg/m**^**2**^**]**	**WHR**	**Systolic blood pressure [mmHG]**	**Diastolic blood pressure [mmHG]**	**Heart rate [bpm]**
**Placebo group**								
Mean	26.64	1.81	83.68	25.45	0.86	127.64	71.08	69.16
SE	1.48	0.02	2.26	0.56	0.01	2.08	1.64	1.89
N	25	25	25	25	24	25	25	25
**PAS 200 group**								
Mean	25.92	1.82	84.8	25.59	0.86	131.68	73.64	73.32
SE	1.32	0.02	3.11	0.73	0.01	1.5	2.11	2.49
N	25	25	25	25	25	25	25	25
**PAS 400 group**								
Mean	26.48	1.83	85.2	25.59	0.87	129.12	73.56	71.2
SE	1.37	0.01	2.32	0.8	0.01	2.52	2.39	2.84
N	25	25	25	25	25	25	25	25
**Group differences**^**a**^								
χ^2^	0.077	0.250	0.684	0.252	0.550	2,717	1,011	1,758
df	2	2	2	2	2	2	2	2
p	0.962	0.882	0.711	0.882	0.760	0.257	0.603	0.415

^a^based on Kruskal Wallis H test.

PAS 200 group: 200 mg PA & 200 mg PS per day; PAS 400 group: 400 mg PA & 400 mg PS per day.

PA = phosphatidic acid, PS = phosphatidylserine, mg = milligrams, m = meter, kg = kilograms, mmHG = millimeter of mercury, bpm = beats per minute, SE = Standard Error, N = Sample Size.

### Compliance

Mean overall compliance was about 89.65% (SE = 1.30%) as assessed by a medication event monitoring system (MEMS®) and about 95.31% (SE = 0.62%) as assessed by counting of returned capsules. Analyses for covariance were performed to explore the potential influence of compliance on outcome measures. Neither MEMS® data nor counting the returned capsules showed an effect of compliance on ACTH, serum cortisol or saliva cortisol response to the TSST (all p > 0.05, data not shown).

### Stress responsivity

Acute stress was induced by the Trier Social Stress Test (TSST). Induction of acute stress was confirmed by significant time effects in repeated measures analyses of variance for relevant variables. As expected, the TSST induced a pronounced increase in salivary cortisol, serum cortisol, and ACTH levels as well as heart rate and a decrease of pulse transit time (PTT) (see Table 2). The PTT is determined by the R wave of the heart beat to the pulse wave arrival at the finger. PTT is used as an index for arterial blood pressure [44-46] and as a biomarker for stress reactivity. Acute stress is associated with an increased blood pressure and a reduced PTT [47,48].

**Table 2:** Biological stress response

	**Heart rate**	**Pulse transit time**	**Saliva Cortisol**	**Serum Cortisol**	**ACTH**
F	130.94	209.66	116.270	116.27	199.22
df, error df	1.98, 124.52	2.74, 147.89	2.10, 128.00	2.10, 128.0	1, 60
p	**< 0.001**	**< 0.001**	**< 0.001**	**< 0.001**	**< 0.001**
η_p_^ 2^	0.675	0.795	0.656	0.656	0.769

Significant p-values are written in bold letters.

ACTH = adrenocorticotropic hormone.

Furthermore, subjects showed an increase of state anxiety (STAI: State-trait-anxiety inventory; VAS: visual analogue scales), perceived stress, insecurity (VAS), and negative mood (MDBF: Multidimensional mood state questionnaire) (Table 3).

**Table 3:** Psychological stress response

	**MDBF**			**STAI**	**VAS**		
	**good mood**	**alertness**	**calmness**	**state anxiety**	**stress**	**anxiety**	**insecurity**
F	42.04	28.22	74.43	64.13	49.96	34.46	67.95
df, error df	1, 65	1, 64	1, 64	1, 65	2, 128	1.81, 117.83	2, 130
p	**< 0.001**	**< 0.001**	**< 0.001**	**< 0.001**	**< 0.001**	**< 0.001**	**< 0.001**
η_p_^ 2^	0.393	0.306	0.538	0.497	0.438	0.346	0.511

Significant p-values are written in bold letters.

STAI = state trait anxiety inventory, MDBF = multidimensional mood state questionnaire, VAS = visual analogue scale.

### Treatment effects

We compared differences between treatment groups (placebo, PAS 200 and PAS 400) and between subjects scoring either high or low on a chronic stress inventory (TICS). To compare general cortisol levels and cortisol increases, we calculated the area under the curve with respect to ground (AUC_G_) and its increase (AUC_I_) as described by Pruessner and colleagues [49]. The AUC_G_ provides information on general cortisol levels whereas the AUC_I_ on cortisol increase and reactivity.

#### Treatment group

Neither supplementation of PAS 200 nor of PAS 400 resulted in significant effects on saliva or serum cortisol levels on the overall study population (saliva cortisol: AUC_G_: χ^2^_  2_ = 0.44, p = 0.801; AUC_I_: χ^2^_  2_ = 2.01, p = 0.366; serum cortisol: AUC_G_: F_2, 61_ = 0.56, p = 0.576; AUC_I_: F_2, 59_ = 2.19, p = 0.121). For ACTH responses there was a significant main effect of treatment group (F_2, 59_ = 3.66, p = 0.032). Tukey post hoc tests revealed significant lower ACTH levels in the PAS 400 group compared to the PAS 200 group (p = 0.025).

#### Chronic stress level

Furthermore, no significant main effect of chronic stress was observed for ACTH increases (F_1, 59_ = 1.820, p = 0.182), AUC_G_ saliva cortisol (Z = −0.28, p = 0.783), and AUC_G_ serum cortisol (F1, 61 = 0.14, p = 0.721). However, high chronically stressed subjects (HCS) showed a tendency towards higher saliva cortisol AUC_I_ (Z = −1.70, p = 0.089) and significantly higher serum cortisol AUC_I_ (F1, 59 = 13.05, p = 0.001) than low chronically stressed subjects (LCS).

#### Subgroup analysis

To further explore the impact of chronic stress levels on treatment effects, we compared subgroups of HCS and LCS.

**Figure 1:**
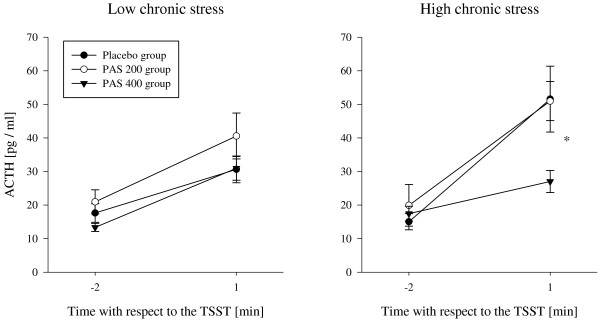
ACTH response to acute stress; *p < 0.05.*PAS 200 group: 200 mg PA & 200 mg PS per day; PAS 400 group: 400 mg PA & 400 mg PS per day.*

**Figure 2:**
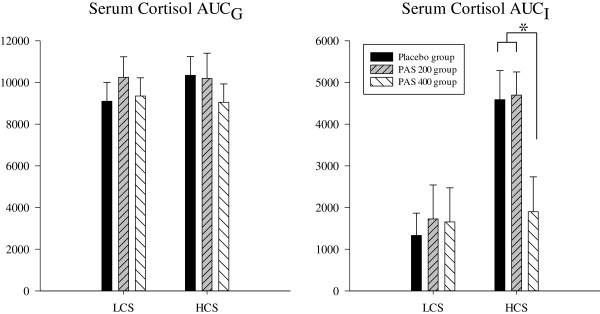
Serum cortisol response to acute stress; *p < 0.05.*PAS 200 group: 200 mg PA & 200 mg PS per day; PAS 400 group: 400 mg PA & 400 mg PS per day.*

**Figure 3:**
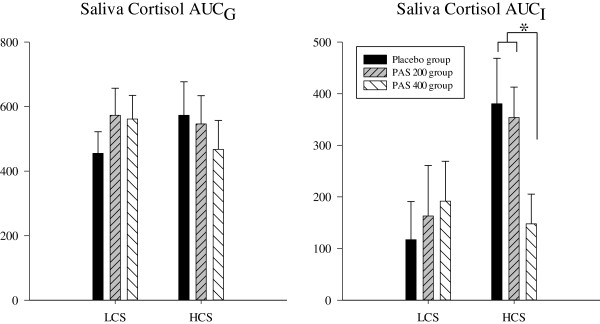
Saliva cortisol response to acute stress; *p < 0.05.*PAS 200 group: 200 mg PA & 200 mg PS per day; PAS 400 group: 400 mg PA & 400 mg PS per day.*

HCS of the PAS 400 group showed significantly lower increases of ACTH, serum cortisol, and saliva cortisol as compared to the placebo and the PAS 200 group. These effects could not be shown in LCS of the PAS 400 group. Also, no differences were observed for HCS and LCS of the PAS 200 group as compared to placebo. This suggests that a supplementation with PAS 400 normalized the enhanced ACTH- and cortisol-responses to an acute stressor in subjects reporting a chronic stress load (Figures 1, 2 and 3). The statistical information on effects of PAS on the endocrine stress response is provided in Table 4 for both HCS and LCS. Comparable to our previous study [27], no effects of supplementation were found for autonomic (heart rate; pulse transit time) and for psychological response measures (mood, insecurity, perceived stress) in both low and high chronically stressed subjects (data not shown). While we observed significant dampening effects on anxiety in the first study, this effect was only marginal in the present study (p < 0.07).

**Table 4:** **Statistical information on group differences for AUC**_**G **_**and AUC**_**I **_**for salivary cortisol, serum cortisol and ACTH levels in response to acute stress**

	**low stressed subjects**			**high stressed subjects**		
**salivary cortisol**^**a**^						
	χ^2^	df	p	χ^2^	df	p
AUC_G_	1.32	2	0.518	0.82	2	0.664
AUC_I_	0.43	2	0.805	6.48	2	**0.039**
*Post hoc testing AUC*_*I*_*in high stressed subjects*^*b*^						
	*Z*	*p*	*r*			
*PAS 400 group vs. Placebo group*	*−1.97*	***0.049***	*0.41*			
*PAS 400 group vs. PAS 200 group*	*−2.37*	***0.018***	*0.51*			
*PAS 200 group vs. Placebo group*	*−0.21*	*0.833*				
**serum cortisol**^**c**^						
	F	df, error df	p	F	df, error df	p
AUC_G_	0.44	2, 31	0.651	0.55	2, 30	0.585
AUC_I_	0.09	2, 31	0.911	4.87	2, 28	**0.015**
*Post hoc testing AUC*_*I*_*in high stressed subjects*^*d*^						
			*p*			
*PAS 400 group vs. Placebo group*			***0.035***			
*PAS 400 group vs. PAS 200 group*			***0.033***			
*PAS 200 group vs. Placebo group*			*0.995*			
**ACTH**^**e**^						
	F	df, error df	p	F	df, error df	p
group difference	1.60	2, 30	0.218	2.43	2, 29	0.106
increase differences	0.31	2, 30	0.737	4.54	2, 29	**0.019**
*Post hoc testing ACTH increase in high stressed subjects*^*e*^						
	*F*	*df, error df*	*p*			
*PAS 400 group vs. Placebo group*	*8.22*	*1, 19*	***0.010***			
*PAS 400 group vs. PAS 200 group*	*8.23*	*1, 20*	***0.009***			
*PAS 200 group vs. Placebo group*	*0.25*	*1, 19*	*0.627*			

PAS 200 group: 200 mg PA & 200 mg PS per day; PAS 400 group: 400 mg PA & 400 mg PS per day.

Significant p-values are written in bold letters. Post hoc analyses are written in italic letters, ACTH = adrenocorticotropic hormone.

^*a*^*statistics based on Kruskal Wallis H Test.*

^*b*^*statistics based on Mann–Whitney U test.*

^*c*^*statistics based on univariate analysis of variance.*

^*d*^*statistics based on Tukey post hoc testing.*

^*e*^*statistics based on repeated measures analysis of variance.*

## Discussion

In the present study, we investigated the effects of a 42-day dose-dependent intake of PAS on biological and psychological responses to the TSST in both HCS and LCS. As expected, the TSST reliably induced robust biological and psychological stress responses. Perceived stress responses were similar in all treatment groups. The placebo group showed a slight tendency for higher anxiety (VAS) during the TSST as compared to the PAS groups. Compliance assessed by MEMS® (about 90%) and by counting remaining capsules (about 95%) was rather high. Differences between both might have been caused by subjects taking their daily dose along to work with only one opening of the track cap which then results in a lower number of total openings. In that case the number of openings as assessed by MEMS® does not reflect the number of intake occasions.

A pronounced stress dampening effect of PAS on HPAA responses could only be observed for the 400 mg treatment group in HCS. These data confirm our previous findings, and are in line with other studies showing that acute and chronic administration of PS and PAS can normalize cortisol responses to acute physical and mental stress [27,30-32,34,35].

As addressed in the introduction, chronic stress can first induce a hyper- and then a subsequent hypo-cortisolemic state. Phospholipid effects on the HPAA can best be described in terms of a normalization of stress responsivity. If chronic stress results in a hyper-responsivity of the HPAA, as observed in this and other studies [15,27], phospholipids can be expected to buffer the HPAA response to stress. On the other hand, phospholipids may restore normal ACTH- and cortisol responses to stress in subjects with a reduced HPAA response [13,28]. Notably, relaxation and stress management techniques show similar effects [50-55]. These observations support the assumption that both phospholipids and relaxation can normalize the HPAA-responsivity to stress.

As in our previous PAS study, effects of PAS 400 could only be observed for endocrine but not for autonomic stress responses. If PAS would primarily affect the stress response network in the brain, one would expect common secondary effects on psychological, endocrine, and autonomic measures. This is unlikely, since in both studies psychological effects were inconsistent and autonomic effects could not be observed. This points to the possibility, that PAS primarily targets peripheral components of the HPAA. Chronic stress has been shown to reduce cortisol binding globulin (CBG) [56-58]. CBG is a glycoprotein synthesized in the liver and secreted in the blood where it binds with a high affinity but low capacity to glucocorticoid hormones, such as cortisol in humans and corticosterone in laboratory rodents. In mammals, 95% of circulating glucocorticoids are bound to either CBG (80%) or albumin (15%), and only the 5% free fraction is able to enter the brain [59]. During stress, the concentration of glucocorticoids rises significantly, and the free fraction increases even more, once CBG becomes saturated. However, glucocorticoids unbound to CBG are cleared from the blood more quickly. It is assumed that CBG plays an important role in the fast actions of glucocorticoids on behavior by maintaining a blood glucocorticoid pool that will be able to access the brain for the fast effects of glucocorticoids [60,61].

The observed pronounced increase of ACTH and cortisol in the TSST can possibly be explained by a drop of CBG in chronically stressed subjects. If so, one may hypothesize that PAS 400 first causes a normalization of CBG levels under such conditions, which then would result in a normalization of the activity and reactivity of the HPAA.

## Conclusions

In chronically stressed subjects, PAS 400 can be expected to buffer a hyper-responsivity of the HPAA to acute stressors by normalizing cortisol responses. In contrast, PAS 400 does not affect endocrine stress response in LCS who do not have elevated cortisol levels. Although sample sizes for subgroup analyses of chronic stress were rather small, the results of this study are intriguing.

The CBG-hypothesis, derived from this study, can now be tested to elucidate a possible relevant mechanism of the action of PAS. If this hypothesis holds true, an indication of PAS for stress related disorders is evident, particularly for disorders which are considered to occur in consequence of stress-elevated cortisol levels, such as cardiovascular disease, central obesity, and the metabolic syndrome [5,62]. In sum, we conclude that PAS 400 (MemreePlus™) is a safe and effective supplement for the reduction of both physical and mental stress. Based on the previous study, it can be speculated that women also benefit from MemreePlus™ supplementation. This needs to be confirmed in further studies.

### Participants and methods

#### Participant selection

**Figure 4:**
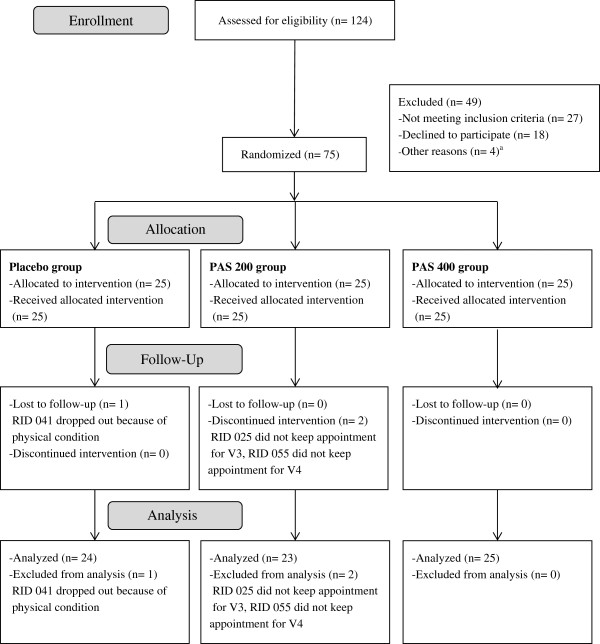
**Subject flow chart.***PAS 200 group: 200 mg PA & 200 mg PS per day; PAS 400 group: 400 mg PA & 400 mg PS per day. *^*a*^*one subject spoke only poor German, three subjects fulfilled the criteria for the high stressed subgroup when no additional subject was needed for this subgroup.*

Healthy, non-smoking male volunteers between age 20 and 45 years were recruited by newspaper advertisement and flyer distribution in the area of Trier, Germany. Subjects’ health was assessed in a structured medical interview by a study physician and by assessing clinical blood chemistry. Individuals were not eligible if any of the following criteria applied: known allergies to ingredients of the test substance, addiction to nicotine, drugs or alcohol, recent changes in nutritional habits (e.g. start of a weight loss diet), any serious general illness within the last 12 months, any febrile illness (longer than 24 hours) within seven days prior to assessment, any intake of antibiotics during the four weeks prior to study inclusion, diabetes mellitus, heart disease, hypertension, kidney disease, significant respiratory disease, or epilepsy, immunologic or infectious disease (e.g. hepatitis, tuberculosis, HIV or AIDS, lupus, rheumatoid arthritis) which could place the subject at risk or interfere with the accuracy of the study results, current or past participation in a TSST study, employee of the sponsor or CRO, medications that are likely to affect treatment response, or any conditions that may affect the ability of the individual to complete the study or the interpretation of the study results.A total number of 124 subjects were screened for study inclusion and exclusion criteria of which 75 male subjects met the eligibility criteria. These subjects were randomly assigned and stratified by chronic stress level to one of the three treatment groups (25 subjects per group: placebo, PAS 200, PAS 400). After study inclusion, one subject of the placebo group and two subjects of the PAS 200 group were lost to follow up, leaving 24 subjects of the placebo group, 23 subjects of the PAS 200 group, and 25 subjects of the PAS 400 group for analysis (total sample size of 72 subjects) (see Figure 4). The study protocol was approved by the ethical review board of the Medical Association of Rhineland-Palatinate (Mainz, Germany), and written informed consent was obtained from all participants. The study was performed in accordance with the ethical principles that have their origin in the Declaration of Helsinki and are consistent with International Conference on Harmonization/Good Clinical Practice (2008).

### Study design and procedures

This was a randomized, double-blind, placebo-controlled, single-center study with three treatment arms (placebo, PAS 200, PAS 400 daily for six weeks). The study included four visits at the study site and had a total duration of 44 days for each subject.

Before subjects were invited to the study site, a first screening took place by telephone interview. Here, subjects received some general information on study procedures as well as a first check of inclusion and exclusion criteria. At visit one (V1), subjects received an extended written information on the aim of this study and all detailed study procedures. After clarifying possible questions, the signed written informed consent was obtained. The study physician then assessed vital signs and took a blood sample for determining safety parameters (i.e. blood profile for hemoglobin, hematocrit, platelet count, leukocytes, monocytes, neutrophils, eosinophils and basophils). Thereafter, subjects completed the Trier Inventory for Chronic Stress (TICS [43]). Chronic stress was determined using the Screening Scale for Chronic Stress, a subscale of the TICS. For classification of low and high chronic stress, the median value of 13 reported by Schulz and colleagues [43] was used as a cut off value. Thirty-five HCS and 35 LCS were randomly assigned to one of the three treatment groups. Randomization sequence was stratified with a 1:1 allocation using random blocks of variable sizes with the computer software SPSS 20.0 (IBM Corporation, Somer NY; USA).

At visit two (V2) those subjects meeting eligibility criteria received a random number. According to the randomization sequence, subjects received their daily supplementation of 4 capsules for the following 21 days. Capsules were packed in a container with a Medication Event Monitoring System (MEMS®) track cap (Aardex Ltd, Zug, Switzerland) to monitor container openings and indicating protocol compliance. MEMS® recorded time and date of each opening electronically. At visit three (V3), MEMS® track caps were read out and subjects received their supplementation dose for the following three weeks. At the final study visit (V4), a post supplementation blood sample for safety parameters was taken and the Trier Social Stress Test (TSST); [63] was performed to induce an acute psycho-social stressful situation. At V2, V3 and V4 subjects were carefully checked for adverse events and concomitant medication.

V4 had an overall duration of 180 minutes. Subjects received their last supplement dosage 90 minutes prior to the stress test. Pre- and post-stress psychometrics tests were performed. For HPAA activity measures saliva (free cortisol) and blood (total cortisol) samples were taken at 2 min. prior to the TSST as well as 1 min., 10 min., 20 min., 30 min. and 60 min. after the TSST. Blood samples, taken at 2 min. prior and 1 min. after the TSST, were also used for ACTH analyses.

Saliva samples were collected using Salivettes® (Sarstedt AG & Co., Nümbrecht, Germany). Blood samples were collected using a vein catheter and 2.7 ml EDTA monovettes® (Sarstedt AG & Co., Nümbrecht, Germany). Saliva samples were stored at −20°C, blood samples were centrifuged and stored at −20°C and −80°C until all participants completed the study.

Saliva cortisol levels were analyzed at the laboratory of the University of Trier, Germany using a competitive solid phase time-resolved fluorescence immunoassay with flouromeric end point detection (DELFIA). The intra- and interassay variability was below 10%.

ACTH was determined using a two-step immunoassay with streptavidin microparticles and electrochemiluminescence detection. Serum cortisol levels were analyzed using an electrochemiluminescence immunoassay (ECLIA). The assay is based on luminescence produced during photochemical reactions in solutions. Both assays used Elecsys® (Roche Diagnostics GmbH, Mannheim, Germany) and their intra- and interassay variability was below 10%.

For measures of the autonomic nervous system, heart rate and pulse transit time (PTT) were measured continuously from 20 min. prior to the TSST to 20 min. after the TSST using an electrocardiogram device (SOMNOscreen™ plus, SOMNOmedics GmbH, Randersacker, Germany). PTT is determined by measuring the electrocardiography and the pulse wave at a finger. Studies indicate that PTT is an appropriate parameter for stress measurement [48,64].

Psychometric assessment included a mood questionnaire MDBF [65], before and after the TSST. State anxiety was assessed with the STAI-X1 [66] before and after the TSST. Furthermore, levels of perceived anxiety, stress and insecurity were measured before, during and after the TSST, as described by Hellhammer and Schubert [67].

### Study treatment

The study supplement PAS (produced by Lipogen Ltd, Israel) and sold as “MemreePlus™” by Lonza Ltd, Switzerland was administered in capsules. Lipogen holds a patent (US 6,410,522 & EP 1201244) and the supplement has an US FDA GRAS (Generally Recognized As Safe) status. In this study, 4 capsules were administered per day: subjects were asked to take two capsules in the morning, one at noon and one in the evening with a glass of water after meals. Single capsules of the PAS 400 group (400 mg PA & 400 mg PS per day) consisted of 100 mg phosphatidylserine (PS) and lysophosphatidylserine, 100 mg phosphatidic acid (PA) and lysophosphatidic acid, 235 mg other phospholipids and glycerides, 5 mg silicone dioxide. Single capsules of the PAS 200 group (200 mg PA & 200 mg PS per day) consisted of 50 mg phosphatidylserine (PS) and lysophosphatidylserine, 50 mg phosphatidic acid (PA) and lysophosphatidic acid, 335 mg other phospholipids and glycerides, 5 mg silicone dioxide. Placebo capsules contained 435 maize starch and 5 mg silicone dioxide. All capsules were identical in size, shape and colour.

### Stress test

The Trier Social Stress Test TSST; [63] was established in 1993 combining key elements of social stress with novelty, unpredictability, uncontrollability and ego-involvement in a standardized laboratory protocol for approximately15 minutes. Since then, it has been used in many clinical studies and in various areas of research. A meta-analysis of stress test protocols of Dickerson and Kemeny [68] showed that the protocol of the TSST is most effective in provoking HPAA responses. Furthermore, the TSST has been shown to provoke a strong increase in an individual’s perception of stress, insecurity and anxiety [67].

The TSST protocol compromises an introduction by the study manager: the subject is introduced to a panel and is asked to prepare for a job interview in a new company. After this introduction, the study manager leaves the TSST-room and the subject had a 3-min. period to prepare for the job interview. The 5-min. interview is followed by a 5 min. mental arithmetic task, stepwise subtracting 17 from 2023 as quickly and correctly as possible. The TSST is terminated by the study manager leading the participant back to every participant’s individual room. All TSSTs were conducted in the afternoon to control for diurnal cycles.

### Statistical analysis

For statistical analyses, we used the software SPSS 21 (IBM Corporation, Somer NY, USA). For statistical inference, different statistical tests were performed. Only two-sided tests were used and data were interpreted on an α-level below 5% (α < .05). Baseline characteristics and demographics as well as data violating normality distribution were analyzed using Kruskal Wallis H test.

For cortisol the area under the curve with respect to ground (AUC_G_) and its increase (AUC_I_) were calculated using the formula described by Pruessner and colleagues [49]. The AUC_G_ holds information about general cortisol levels, as it is an integrated value of all measurements. The AUC_I_ holds information about the increase of cortisol levels, as it is an integrated value of all measurements adjusted for the cortisol level under resting conditions. For salivary cortisol AUC_G_ and AUC_I_ we conducted Kruskal-Wallis-H-tests for effects of treatment groups and Mann–Whitney-U-tests for treatment group to treatment group comparison and for effects of chronic stress level. For serum cortisol AUC_G_ and AUC_I_ we conducted univariate analyses of variance with treatment group and chronic stress level as fixed factors. Post hoc comparison was done with Tukey post hoc testing. For ACTH we conducted repeated measures analyses of variance with treatment group and chronic stress level as factors. Post hoc comparison of treatment groups was done with Tukey post hoc testing. Post hoc comparison of increase differences was done by repeated measures analyses of variance, with analysis-filters for relevant groups.

## Abbreviations

ACTH: Adrenocorticotropic hormone; AUC_G_: Area under the curve with respect to ground; AUC_I_: Area under the curve with respect to its increase; BMI: Body mass index; CBG: Corticosteroid-binding globulin; CRO: Clinical research organization; DELFIA: Dissociation-enhanced lanthanide fluorescence immunoassay; ECLIA: Electrochemiluminescence immunoassay; EDTA: Ethylendiamintetraacetat; HCS: High chronically stressed subjects; HPAA: Hypothalamic-pituitary-adrenal axis; LCS: Low chronically stressed subjects; MDBF: Multidimensional mood state questionnaire (German: Mehrdimensionaler Befindlichkeitsfragebogen); MEMS: Medication event monitoring system; PA: Phosphatidic acid; PS: Phosphatidylserine; PAS: Phosphatidic acid and phosphatidylserine; PTT: Pulse transit time; SE: Standard error; SPSS: Statistical package for the social sciences; STAI: State trait anxiety inventory; TICS: Trier inventory for chronic stress; TSST: Trier social stress test; V: Visit; VAS: Visual analogue scale; WHR: Waist to hip ratio.

## Competing interests

This study was financed by Lipogen Ltd (DR) and Lonza Ltd (UF) and performed by Daacro GmbH & Co.KG (JH, DV, NF), a clinical research organization. DR, UF disclose a commercial interest. JH, DV, NF declare that they have no competing interest.

## Authors’ contributions

JH, UF, DR designed the clinical study protocol. NF managed the study. DV performed the statistical analysis. JH, DV, NF drafted the manuscript. All authors read and approved the final manuscript.
